# A Review of Danshen Combined with Clopidogrel in the Treatment of Coronary Heart Disease

**DOI:** 10.1155/2019/2721413

**Published:** 2019-02-19

**Authors:** Zhaojian Zhang, Yu Wang, Wangxiao Tan, Siwei Wang, Jinghua Liu, Xiao Liu, Xiaoying Wang, Xiumei Gao

**Affiliations:** ^1^Key Laboratory of Formula of Traditional Chinese Medicine (Tianjin University of Traditional Chinese Medicine), Ministry of Education, China; ^2^College of Traditional Chinese Medicine, Tianjin University of Traditional Chinese Medicine, Tianjin 301617, China

## Abstract

**Objective:**

Danshen, the root of* Salvia miltiorrhiza* Bunge, is a traditional herbal medicine in China, which has been used to treat irregular menstruation, cold hernia, and abdominal pain for thousands of years. Danshen is frequently used in combination with drugs to treat cardiovascular diseases. Clopidogrel is a commonly used drug for treating coronary heart disease, but clopidogrel resistance restricts its development. Therefore, the clinical efficacy of Danshen combined with clopidogrel treats coronary heart disease and the relationship between Danshen and clopidogrel metabolism enzymes is suggested for future investigations.

**Materials and Methods:**

The information was collected by searching online databases, and the RevMan 5.3 software was used to perform meta-analysis.

**Results:**

Twenty-two articles, including 2587 patients, were enrolled after the evaluation. Meta-analysis showed that Danshen combined with clopidogrel was more effective than clopidogrel alone in treating coronary heart disease by improving clinical curative effect, reducing the frequency of angina pectoris, improving electrocardiogram results, shortening the duration of angina pectoris, and easing adverse reactions. Danshen inhibited carboxylesterase 1 and most enzyme of cytochrome P450, especially cytochrome P450 1A2, which may affect the metabolism of clopidogrel.

**Conclusion:**

Danshen combined with clopidogrel may compensate for individual differences of clopidogrel resistance among individuals in the treatment of coronary heart disease. Meanwhile, the inhibitory effect of Danshen on cytochrome P450 and carboxylesterase 1 could be partly responsible for the synergistic and attenuating effects of Danshen combined with clopidogrel.

## 1. Introduction

Danshen (Salviae miltiorrhizae, Chinese sage, Radix Salviae miltiorrhiza, red sage, danshen, Tan Shen) is the root and rhizome of* Salvia miltiorrhiza* Bunge. Danshen, a traditional herbal medicine in China, had been used to treat irregular menstruation, cold hernia, and abdominal pain, because the theory of traditional Chinese medicine holds that it activates blood circulation to dissipate blood stasis, calming, and relieving pain [1]. Today, Danshen has proved effective in organ protection, brain protection, and antitumour action, and the most widely used is in the field of cardiovascular disease. Researchers proved that, in China, nearly all ( 99% ) hospitals used early intravenous traditional herbal medicine for acute myocardial infarction, and Danshen accounts for the first three of these herbs. Patients receiving early intravenous herbal medicine may have fewer cardiovascular risk factors [2]. Meta-analysis showed that Danshen depside salts, extracted (as dry extract, refined) from the dried root of Danshen Bunge, combined with conventional treatment, were superior to conventional treatment alone in improving angina symptoms; bioinformatics analysis found that Danshen depside salts may target* jun, tnf, nfkb, fos, and bcl-2* exerts effects against cardiovascular disease [3, 4]. In view of the antioxidant [5], anti-inflammatory [6], antiapoptotic [7], and cardioprotective effects [8] of Danshen, it is often used in combination with other drugs. Atorvastatin is an effective lipid-lowering drug without mentioning its side effects. Studies showed that atorvastatin combined with Danshen and Pueraria lobata yielded stronger hypolipidemic effects and fewer side effects than atorvastatin used alone [9]. In terms of prevention and treatment of thrombotic diseases, Danshen is also used in combination with other drugs: the combination of Danshen and aspirin can more effectively treat patients with coronary heart disease (CHD), especially those with diabetes or hyperlipidaemia [10]; Danshen can enhance the antiplatelet effect of clopidogrel by prolonging bleeding time of coagulation parameters, restraining arteriovenous bypass thrombosis [11].

Clopidogrel, as an adenosine diphosphate receptor antagonist, is widely used in cardiocerebrovascular disease. Researchers found that clopidogrel can effectively improve the clinical symptoms and haemodynamic indexes of patients with CHD by reducing the platelet aggregation rate and decreasing the inside thrombosis, thus improving microvascular endothelial function. However, researchers have also revealed that clopidogrel has individual differences in the treatment of CHD. Some researchers declared that this situation may be related to clopidogrel resistance or high on-treatment platelet reactivity, and evidence showed that older patients were more likely to be having high on-treatment platelet reactivity and resistance to clopidogrel [12, 13]. For those with low clopidogrel response, clopidogrel was for the most part overdosed, which appeared to be ineffective in branch atherosclerotic diseases [14, 15] or was switched to other drugs such as prasugrel or ticagrelor or used in combination with other medications [16, 17]. Research illustrated that clopidogrel combined with aspirin caused a lower recurrence rate of coronary artery disease in the first year after coronary artery bypass grafting compared to the addition of aspirin to placebo, despite aspirin and clopidogrel as classic antiplatelet drugs commonly used in combination for CHD patients [18, 19]. Meanwhile, clopidogrel is often combined with proton pump inhibitors; however, the clinical significance of the interaction between clopidogrel and proton pump inhibitors (PPIs) remains unclear [20].

Clopidogrel, as a prodrug, is metabolized by several metabolic enzymes including carboxylesterase 1 (CES1) [51] and cytochrome P450 (CYP450) [52]. The majority of the absorbed clopidogrel dose never enters the bioactivation cascade since more than 85% of the parent compound could be hydrolyzed by CES1 to its major inactive carboxylic acid metabolite (clopidogrel carboxylic acid), whereas the remainder (about 15%) is oxidized to the intermediate metabolite 2-oxo-clopidogrel by the hepatic CYP [53]. Tanshinones, phenanthrene-quinone derivatives isolated from traditional Chinese herbal Danshen, have been found to be potent inhibitors of both CES1 and CES2 [54–56]. Thus, Danshen may improve the efficacy of clopidogrel on angina pectoris by improving the bioavailability of clopidogrel. However, Danshen is composed of many compounds, and the different effects of its active components on clopidogrel-related metabolic enzymes may change this conclusion, so it is necessary to find out the relationship between them.

In this article, the meta-analysis based on randomized controlled trials (RCTs) about Danshen combined with clopidogrel in the treatment of CHD was conducted to provide substantiation of evidence-based medicine, and we discussed the effect of Danshen on CES1 and CYP450.

## 2. Meta-Analysis of Danshen Combined with Clopidogrel Based on CHD

The meta-analysis, which was conducted on randomized controlled trials of Danshen and clopidogrel in the treatment of CHD from 1966 to 2018, was used to evaluate the efficacy of Danshen and clopidogrel combination therapy systematically in the treatment of CHD.

### 2.1. Methods

#### 2.1.1. Inclusion Criteria

The inclusion criteria consisted of (1) randomized controlled clinical trials, no restrictions on blindness and language; (2) patients with a clinical diagnosis of CHD, with clear diagnostic criteria referred to in the paper; (3) control groups that had clopidogrel, with clearly stated dosage, and were permitted to use conventional treatment such as nitrates, statins, aspirin in small doses, and so on; treatment groups that were administered the same drugs as control groups but with Danshen (forms not limited) added; (4) unrestricted drug dose and treatment course; (5) one of the following results that was included in the study: total effectiveness on CHD or electrocardiogram (ECG) efficacy.

#### 2.1.2. Exclusion Criteria

The exclusion criteria consisted of (1) nonrandomized controlled studies (reviews, animal studies, or case–control studies); (2) controls that were not given clopidogrel; (3) study of identical data or repeated publication.

#### 2.1.3. Retrieval Strategy

Literature retrieval was conducted using electronic retrieval methods. Search databases included China Knowledge Resource Integrated (CNKI) database (1979 to April 2018), Chinese Science and Technique Journals (VIP) database (1989 to April 2018), Wan Fang (Wan Fang) database (1990 to April 2018), Cochrane Library (1999 to April 2018), Web of Science (1995 to April 2018), and PubMed (1966 to April 2018). Subject words in Chinese retrieval were: All fields “Danshen”; “clopidogrel”; “coronary heart disease”; “Angina pectoris”. Subject words in English retrieval were: All fields (“Danshen" [MeSH] OR Tan Seng OR Dan-Shen OR Dan Shen OR Chinese Salvia OR Chinese Salvias OR Salvia, Chinese OR Salvias, Chinese OR Danshene OR Danshen) AND “clopidogrel" AND (“Coronary heart disease" [MeSH] OR Coronary Diseases OR Disease, Coronary OR Diseases, Coronary OR Coronary Heart Disease OR Coronary Heart Diseases OR Disease, Coronary Heart OR Diseases, Coronary Heart OR Heart Disease, Coronary OR Heart Diseases, Coronary) in Clinical Trial. Search years for publication were to 2018.

#### 2.1.4. Data Extraction and Management

Data concerning details about the participants, intervention, and outcomes were extracted independently by two reviewers (Zhaojian Zhang and Yu Wang). The data extraction form included the following items: (1) general information: title, authors, and year of publication; (2) intervention: drug intervention of control groups and treatment groups; (3) patients: total number and number in control groups and treatment groups; (4) outcomes for all control groups and treatment groups: the overall effective rate, the number in the overall effective rate, the result of the efficacy evaluation index, and adverse reactions.

#### 2.1.5. Quality Evaluation

Methodological quality assessments included in the literature were evaluated by 2 researchers (Wangxiao Tan and Siwei Wang) in accordance with standards provided in the Cochrane Handbook for Systematic Review of interventions [57]. The evaluation included random sequence generation, allocation concealment, blinding of participants and personnel, blinding of outcome assessment, incomplete outcome data, and selective reporting and other bias. The quality of all studies included in the literature was categorised according to low risk of bias, high risk of bias, and unclear risk of bias. Any disagreements were resolved by mutual consensus.

#### 2.1.6. Data Synthesis

Revman 5.3 software provided by the Cochrane Collaboration was used for data analyses. For dichotomous studies, the pooled odds ratio (OR) with 95% confidence interval (CI) was used as the effect measure. For the continuous outcome, the weighted mean difference (WMD) was used as the effect measure. The number, age, gender, species of Danshen, dose of clopidogrel, dose of Danshen, course of disease, and time of treatment included in the RCTs were analysed to determine whether it was heterogeneous. As long as I^2^ was no greater than 50%, the heterogeneity could be accepted. The meta-analysis was performed using a random-effects model or a fixed-effects model based on heterogeneity. Random-effects model analysis was used when obvious heterogeneity exists. When no heterogeneity was present, fixed-effects model analysis was used.

### 2.2. Result

#### 2.2.1. Literature Search Results

**Figure 1:**
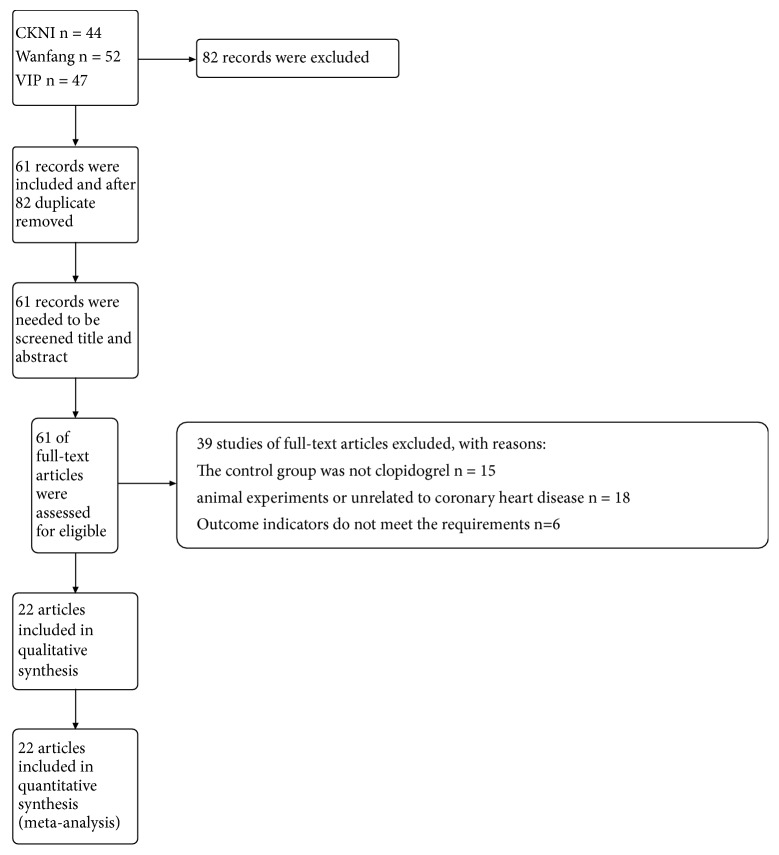
Literature screening process.

In total, 143 papers were retrieved from a number of search libraries. Eighty-two articles were excluded and 61 articles were extracted by removing the same articles from different search libraries and those published by the same author. Through full-text reading, 61 articles continued to be screened. Only 22 articles were ultimately included. The detailed process of search and identification is shown in Figure 1.

#### 2.2.2. Trial Characteristics

Twenty-two RCTs were included, covering a total of 2587 patients with CHD, including 1324 cases in the experimental group and 1263 cases in the control group. The largest sample size of a single RCTs was 520 cases, and the minimum was 60 cases, with an average of 122 cases. The shortest duration of intervention was 0.2 months and the longest was 12 months. The publication dates of the literature were between 2010 and 2018, and all the experiments were completed in China. Each study reported the general situation of the patient, including age, gender, and severity of the disease, and the baseline of each document was comparable. The feature tables included in the literature are shown in Tables 1 and 2.

#### 2.2.3. Methodological Quality

**Figure 2:**
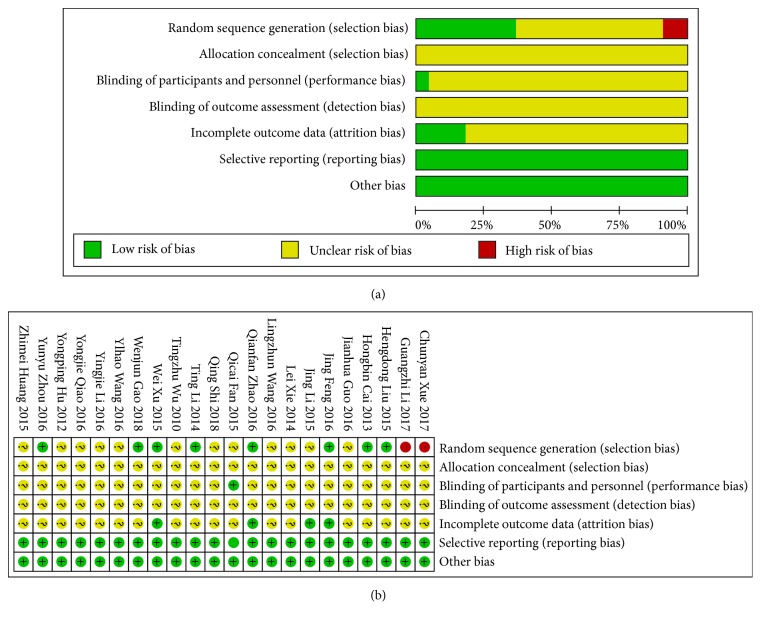
Quality evaluation of literature. (a) Bias risk analysis of literature; (b) bias risk summary.

All RCTs included in the study were limited in terms of research design and methodological information, and methodological quality was low. The methodological quality evaluation was carried out according to the Cochrane Reviewer's Handbook 5.2 risk assessment tool, and the specific evaluation included in the literature is shown in Figure 2.

As shown in Figure 2, (1) for random sequence generation, 8 papers reported methods for generating random sequences; 12 papers used random methods but did not specify the actual implementation, 2 papers divided patients into two groups according to the order of admission. (2) For allocation concealment, 3 papers used random methods but did not specify the actual implementation. (3) For blinding of participants and personnel, only one study adopted single blinding; no others mentioned blinding. (4) For blinding of outcome assessment, none of these mentioned blinding of outcome assessment. (5) For incomplete outcome data, only 4 mentioned withdrawals and losses to follow-up, but none of the studies performed intention-to-treat analysis. (6) For selective reporting, we believed all included studies to be free of selective reporting because the same outcomes were described in the methods and reported in the results. (7) For other biases, in all studies the characteristics of participants in different treatment groups were similar at baseline (age, sex), so we considered all included trials to be free of other potential sources of bias.

#### 2.2.4. Effects of Interventions

**Figure 3:**
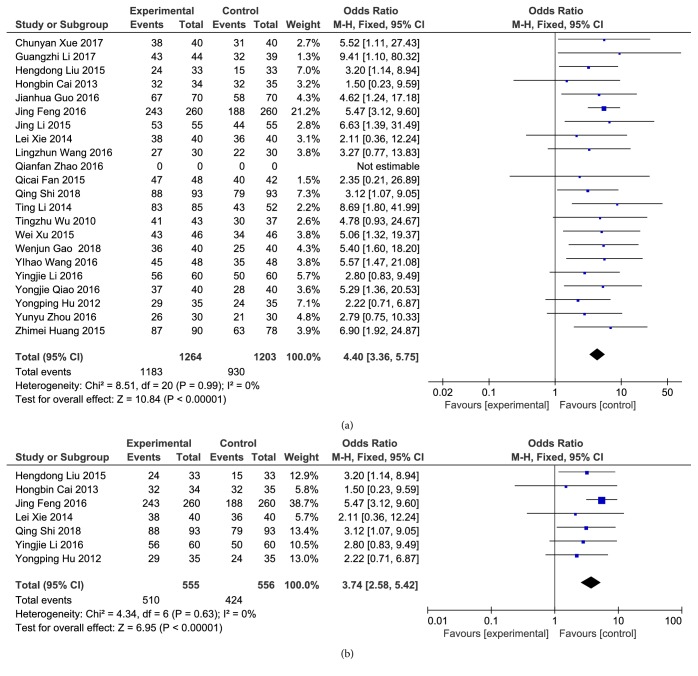
Total effective rate of CHD. (a) Total effective rate of CHD; (b) total effective rate against unstable angina pectoris.

*(1) Total Effective Rate against CHD*. Twenty-one articles reported the total effective rate against CHD after treatment. There was no significant heterogeneity among the studies (P = 0.99, I^2^ = 0%), and the fixed-effects model was used for analysis. The results of meta-analysis showed that compared to clopidogrel alone, combination with Danshen could significantly improve the clinical efficacy on CHD, and differences were statistically significant [Z = 10.84, P <0.00001, OR = 4.40, 95% CI: 3.36, 5.75, as shown in Figure 3(a)].

Among these 21 articles, 7 articles reported the total effective rate against unstable angina pectoris. There was no significant heterogeneity among these 7 studies (P = 0.63, I^2^ = 0%), and the fixed-effects model was used for analysis. The results of meta-analysis showed that compared to clopidogrel alone, combination with Danshen could significantly improve the clinical efficacy against unstable angina pectoris, and differences were statistically significant [Z = 6.95, P <0.00001, OR = 3.74, 95% CI: 2.58, 5.42, as shown in Figure 3(b)].

**Figure 4:**
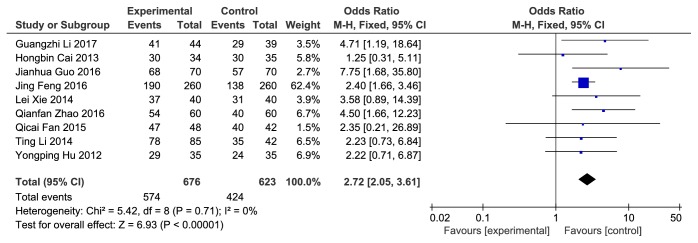
ECG evaluation of CHD.

*(2) ECG Evaluation of CHD. *Nine articles reported the ECG effective rate. There was no significant heterogeneity among these studies (P = 0.71, I^2^ = 0%), and the fixed-effects model was used for analysis. The results of meta-analysis showed that compared to clopidogrel alone, combination with Danshen could significantly improve the ECG rate, and differences were statistically significant [Z = 6.93, P <0.00001, OR = 2.72, 95% CI: 2.05, 3.61, as shown in Figure 4].

**Figure 5:**
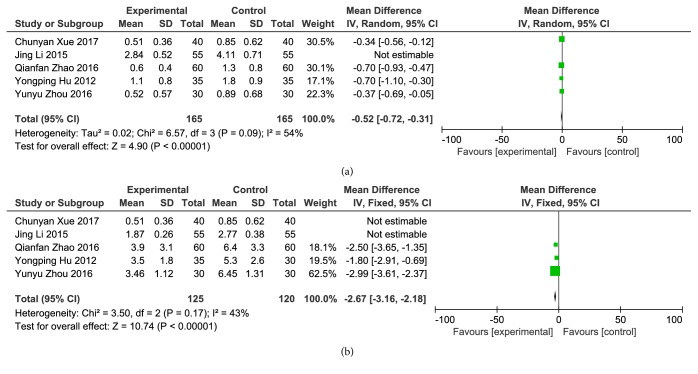
Frequency and duration of angina pectoris. (a) Frequency of angina pectoris; (b) duration of angina pectoris.

*(3) Frequency and Duration of Angina Pectoris*. Five articles reported the frequency of angina pectoris. There was statistical heterogeneity in each study (P <0.00001, I^2^ = 89%), which could not be excluded even after using sensitivity analysis to exclude one article that might have caused heterogeneity; therefore, the random-effects model was used for analysis. The results of meta-analysis showed that compared to clopidogrel alone, combination with Danshen could significantly decrease the frequency of angina pectoris, and differences were statistically significant [Z = 4.90, P <0.00001, MD = –0.52, 95% CI: –0.72, –0.31, as shown in Figure 5(a)].

Five articles reported the duration of angina pectoris. There was statistical heterogeneity in each study (P <0.00001, I^2^ = 95%), which was excluded after using sensitivity analysis to exclude 2 articles that might have caused heterogeneity (P = 0.17, I^2^ = 43%), and the fixed-effects model was used for analysis. The results of meta-analysis showed that compared to clopidogrel alone, combination with Danshen could significantly decrease the duration of angina pectoris, and differences were statistically significant [Z = 10.74, P <0.00001, OR = –2.67, 95% CI: –3.16, –2.18), as shown in Figure 5(b)].

**Figure 6:**
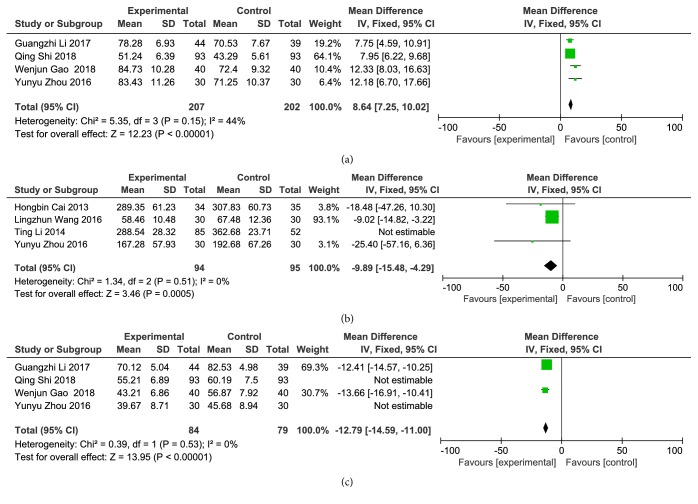
Blood index. (a) Changes in NO; (b) changes in TXB_2_; (c) changes in ET-1.

*(4) Blood Index. *Four articles reported changes of nitric oxide (NO) after treatment. There was no significant heterogeneity among the studies (P = 0.15, I^2^ = 44%), and the fixed-effects model was used for analysis. The results of meta-analysis showed that compared to clopidogrel alone, combination with Danshen could significantly increase the release of NO, and differences were statistically significant [Z = 12.23, P <0.00001, OR = 8.64, 95% CI: 7.25, 10.02, as shown in Figure 6(a)].

Four articles reported changes in thromboxane B2 (TXB_2_) after treatment. There was statistical heterogeneity in each study (P <0.00001, I^2^ = 98%). Statistical heterogeneity was excluded after using sensitivity analysis to exclude 1 article that might have caused heterogeneity (P = 0.51, I^2^ = 0%), and the fixed-effects model was used for analysis. The results of meta-analysis showed that compared to clopidogrel alone, combination with Danshen could significantly reduce the release of TXB_2_, and differences were statistically significant [ Z = 3.46, P = 0.0005, OR = –9.89, 95% CI: –15.48, –4.29), as shown in Figure 6(b)].

Four articles reported changes in endothelin-1 (ET-1) after treatment. There was statistical heterogeneity in each study (P <0.00001, I^2^ = 91%), which was excluded after using sensitivity analysis to exclude 2 articles that might have caused heterogeneity (P = 0.53, I^2^ = 0%), and the fixed-effects model was used for analysis. The results of meta-analysis showed that compared to clopidogrel alone, combination with Danshen could significantly reduce the release of ET-1, and the differences were statistically significant [Z = 13.95, P <0.00001, OR = –12.79, 95% CI: –14.59, –11.00), as shown in Figure 6(c)].

**Figure 7:**
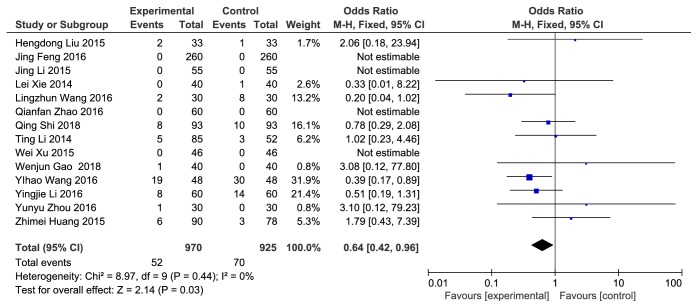
Adverse reactions.

*(5) Adverse Reaction*. Fourteen articles reported adverse reactions. There was no significant heterogeneity among the studies (P = 0.44, I^2^ = 0%), and the fixed-effects model was used for analysis. The results of meta-analysis showed that compared to clopidogrel alone, combination with Danshen could reduce side effects [Z = 2.14, P = 0.03, OR = 0.64, 95% CI: 0.42, 0.96, as shown in Figure 7].

The results proved that Danshen combined with clopidogrel was more effective than clopidogrel alone in the clinical effect on CHD (21 articles [Z = 10.84, P <0.00001, OR = 4.40, 95% CI: 3.36, 5.75]. In addition, this combination proved to be more effective than clopidogrel alone in reducing the rate of unstable angina pectoris (7 articles [Z = 6.95, P <0.00001, OR = 3.74, 95% CI: 2.58, 5.42]), improvement in ECG results (9 articles [Z = 6.93, P <0.00001, OR = 2.72, 95% CI: 2.05,3.61]), in reducing the frequency of angina pectoris (5 articles [Z = 4.90, P <0.00001, MD = –0.52, 95% CI: –0.72, –0.31]), in shortening the duration of angina pectoris (5 articles [Z = 10.74, P <0.00001, OR = –2.67, 95% CI: –3.16, –2.18]), in promoting changes in NO after treatment (4 articles [Z = 12.23, P <0.00001, OR = 8.64, 95% CI: 7.25, 10.02]), in promoting changes in TXB_2_ after treatment (4 articles [Z = 3.46, P = 0.0005, OR = –9.89, 95% CI: –15.48, –4.29]), in promoting changes in ET-1 after treatment (4 articles [Z = 13.95, P <0.0001, OR = –12.79, 95% CI: –14.59, –11.00)], and in reducing adverse reactions (14 articles [Z = 2.14, P = 0.03, OR = 0.64, 95% CI: 0.42, 0.96]).

## 3. Effect of Danshen on the Metabolic Enzymes of Clopidogrel

Drug-induced hepatotoxicity, as the main factor in the recall of drugs, is getting mounting attention, and there are some concerns about drug-induced liver injury with Chinese herb medicine [58, 59]. Clopidogrel, as a prodrug, is metabolized by CES1, CYP450. Research showed that Danshen had almost no liver toxicity yet [60]; on the contrary, tanshinone IIA extract from Danshen can protect against acetaminophen-induced hepatotoxicity [61, 62]. Moreover, Danshen may achieve synergistic effect by increasing the bioavailability of clopidogrel. However, the effect of a single herbal medicine active ingredient on clopidogrel metabolising enzymes may be dissimilar from other single herbal medicines or compound prescriptions on enzymes, such as when water extraction of Danshen or cryptotanshinone has a negligible inhibitory effect on CYP2C19, but dihydrotanshinone has a strong inhibitory effect on it, in addition [43]. Therefore, to determine the interaction between Danshen and clopidogrel metabolising enzymes has clinical guiding role.

Among these metabolic enzymes, carboxylesterase is a multimeric protein that catalyses the hydrolysis of esters, sulfate, and amides. Carboxylic acid metabolite of clopidogrel would discharge in vitro through the CES1 pathway; therefore, the bioavailability of clopidogrel can be improved by inhibiting the liver CES1 [55]. M. Jason Hatfield et al. believe that Danshen has potent human carboxylesterase inhibition by the presence of Tanshinones, and their study shows that the Ki of tanshinone IIA, cryptotanshinone, tanshinone I, and miltirone was 6.89 *μ*M, 0.54 *μ*M, 26.25 *μ*M, and 2.5 *μ*M, respectively [63], which indicated that Danshen may influence the metabolism of clopidogrel to some extent when they are used together [64].

**Figure 8:**
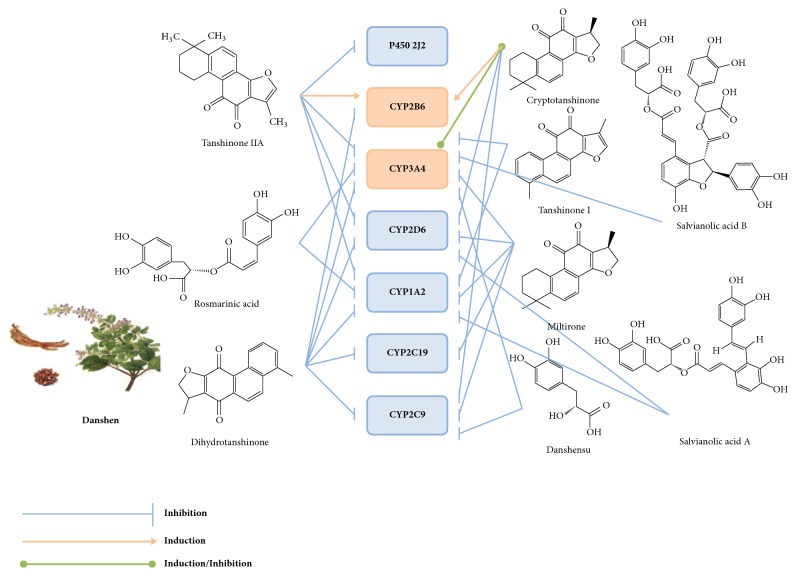
The principal CYP isoforms regulated by active components of Danshen.

CYP450 enzymes are mainly divided into 3 major subfamilies, CYP1, CYP2, and CYP3, according to their gene sequences and homology. Some research has shown that CYP2C19*∗*2 and CYP2C19*∗*17 polymorphism may influence the effect of clopidogrel treatment [65], which provides a new suggestion for the treatment of clopidogrel resistance [66]. The principal CYP isoforms regulated by active components of Danshen are shown in Figure 8. Meanwhile, Table 3 shows half maximal inhibitory concentration (IC_50_) between Danshen's effective components with the CYP450 enzyme system. By the way, in accordance with Kong et al. [67] the inhibitory potency could be classified according to its IC_50_ values, as follows: potent if IC_50_ is ≦20 *μ*g/mL (10 *μ*M); moderate if IC_50_ is 20–100 *μ*g/mL (10–50 *μ*M); and weak if IC_50_ is ≥100 *μ*g/mL (50 *μ*M). Through this classification, we simply define the inhibition or induction of Danshen on CYP450 enzyme system.

CYP1A2 is one of the complex functional oxidase systems of CYP450. Studies have shown that CYP1A2 is an indispensable part of the transformation of clopidogrel into active metabolites [68]. Researches showed that cryptotanshinone, dihydrotanshinone, salvianolic acid A, and miltirone have potential inhibitory effects on CYP1A2, with IC_50_ of 0.75 *μ*M to 3.06 *μ*M, 0.5 *μ*M to 2.25 *μ*M, 5.37 *μ*M and 1.73 *μ*M respectively [44–46, 49]. Besides, the IC_50_ value of rosmarinic acid on CYP1A2 was 10.32 *μ*M, which indicated that rosmarinic acid had moderate inhibitory effect on CYP1A2 [45]. Meanwhile, evidences have revealed that the inhibitory effect of tanshinone IIA and tanshinone I on CYP1A was between potential and moderate, and the IC_50_ value was between 1.3 *μ*M and 10.10 *μ*M, 0.75 *μ*M and 11.61 *μ*M [45–47].

CYP2B6, as a member of the cytochrome P450 family, participates in the metabolism of substances including artemisinin and ketamine and can be inactivate by clopidogrel [69, 70]. Among all the constituents of Danshen, only dihydrotanshinone showed potential inhibitory effect, while tanshinone IIA and cryptotanshinone showed promoting effect [45, 71].

CYP2C9, which accounts for about 20% of the total P450 protein in the liver, is an important member of the second subfamily of CYP450 [72]. In addition, CYP2C9 participates in the metabolism of clopidogrel and attenuates the platelet inhibition mediated by clopidogrel [73]. Furthermore, the gene frequency of CYP2C9 varies greatly among different races and nationalities. Studies have shown that CYP2C9*∗*1 may affect the metabolism of meloxicam [74]. CYP2C9*∗*2 and CYP2C9*∗*3 may be associated with bleeding complications caused by low-dose warfarin [75]. Studies have shown that cryptotanshinone and dihydrotanshinone both have potential inhibitory effects on CYP2C9*∗*1, CYP2C9*∗*2, and CYP2C9*∗*3 [48]. Nevertheless, cryptotanshinone has moderate inhibitory effects on CYP2C9 [44, 47]. Besides, miltirone has potential inhibitory effects on CYP2C9 with IC_50_ values of 8.61 *μ*M [49]. As for danshensu and dihydrotanshinone, they showed moderate and potential inhibitory effects on CYP2C9, respectively [44, 47].

At present, the relationship between CYP2C19 and clopidogrel is still controversial. Studies have shown that the incidence of cardiac complications in CYP2C19 genotype population after taking clopidogrel is significantly higher than that in normal population, but some experiments have shown that no significant difference between CYP2C19 and clopidogrel has been found in the course of treatment [76, 77]. The IC_50_ values of dihydrotanshinone and miltirone for CYP2C19 were 0.6 *μ*M and 26.9 *μ*M, respectively, suggesting that the inhibitory effect of dihydrotanshinone is more potential [43].

As one of the metabolic enzymes of clopidogrel, CYP2D6 is highly expressed in liver and central nervous system. Studies show that dihydrotanshinone, salvianolic acid A, and miltirone have moderate inhibitory effects on CYP2D6, with IC_50_ values of 11.70 *μ*M to 35.4 *μ*M, 11.53 *μ*M and 30.20 *μ*M, respectively [45, 49, 50]. However, two studies showed that tanshinone IIA had a great difference in the effect of CYP2D6. The IC_50_ value of tanshinone IIA on CYP2D6 was 13.47 *μ*M measured by Wen Xu et al. [45]. Another Research by Furong Qiu et al. showed that the IC_50_ value was more than 200 *μ*M [47].

CYP3A4 is a member of the cytochrome P450 oxidase family and participates in the metabolism of most drugs. Dorota Danielak et al. found that CYP3A4*∗*1G had no significant effect on clopidogrel resistance; however Rui Liu et al. believed that CYP3A4*∗*1G might prevent clopidogrel resistance [76, 78]. However, CYP3A4 does play a key role in clopidogrel metabolism. Among several chemical constituents of Danshen, dihydrotanshinone and rosmarinic acid have potential inhibitory effects on CYP3A4 with IC_50_ values of 0.367 *μ*M to 3.22 *μ*M and 5.43 *μ*M, respectively [44, 45]. Meanwhile, for CYP3A4, the IC_50_ values of salvianolic acid B and milton were 12.35 *μ*M and 33.88 *μ*M, respectively, which belonged to the moderate inhibition effect [45, 49], although a study has shown that salvianolic acid B has no significant inhibition on CYP3A4 [47]. Moreover, danshensu and tanshinone I do not have specific IC_50_ values based on CYP3A4 at present, but experiments show that both have inhibitory effects on CYP3A4 [79]. Experiments have shown that cryptotanshinone and tanshinone IIA can induce CYP3A4 [80].

CYP2J2 not only plays an important role in the field of cardiovascular function and cancer, but also serves as an important barrier for intestinal metabolism of many drugs. At present, only one literature reported that tanshinone IIA had potential inhibitory effect on P450 2J2 in all the chemical constituents of Danshen [81].

## 4. Conclusions

This meta-analysis showed that Danshen combined with clopidogrel can improve the clinical efficacy and show the curative effects on electrocardiogram; reduce the frequency and duration of angina pectoris; regulate the level of NO, TXB2, and ET-1; reduce side effects in patients with CHD; and yield more advantages than with clopidogrel alone. However, according to the risk bias table, the quality of the literature included was low, and all the studies were RCTs, but not all the literature had a detailed description of random methods. These problems may lead to greater bias; the included literature is not followed up and therefore cannot be judged. Long-term efficacy may overestimate the effect of combination therapy. Therefore, the conclusions should be interpreted with care until more high-quality RCTs have been published.

Researches show Danshen has both inhibitory and inductive effects on CYP450 enzyme and has a strong inhibitory effect on CES1. Authors believe Danshen combined with clopidogrel could result in clopidogrel hydrolysis partially blocked, while the plasma levels of both clopidogrel and its active metabolite activated by CYPs would be elevated and thus bring better therapeutic effects. Although study showed that the content of tanshinone IIA, tanshinone IIB, cryptotanshinone, etc. in Danshen was limited due to its low bioavailability, so the pharmacokinetic effect of Danshen on clopidogrel in vivo might be slight [82].

In general, clopidogrel is a star drug in the cardiovascular field, but various reports of its resistance restrict its development. Danshen combined with clopidogrel has shown the potential of synergism and attenuation, and the study of the combination of Danshen and clopidogrel in the treatment of coronary heart disease should be more carried out.

## Figures and Tables

**Table 1:** The feature Table 1 of meta-analysis.

Study	Number (Exp/Con)		Gender (Exp/Con)		Age (Exp/Con)		Course of disease/ year (Exp/Con)	
Qing Shi 2018[21]	93	93	50/43	54/39	60.21±3.97	59.43±3.65	-	-
Wenjun Gao 2018[22]	40	40	24/16	22/18	64.92±6.40	65.28±6.39	6.18 ±1.23	6.20 ±1.21
Chunyan Xue 2017[23]	40	40	28/12	26/14	61.35±2.64	61.42±2.71	-	-
Guangzhi Li 2017[24]	44	39	32/12	27/12	71.05±3.83	72.67±4.17	15.12±3.95	14.26 ± 4. 97
Jianhua Guo 2016[25]	70	70	43/27	46/24	77.60±5.40	76.80±5.30	9.20±0.30	11.50±0.50
Jing Feng 2016[26]	260	260	165/95	157/103	63.88±2.85	63.85±2.79	5.88±4.70	5.81±2.79
Qianfan Zhao 2016[27]	60	60	32/28	33/27	53.25±7.15	54.60±7.25	6.34±2.79	6.53±2.68
Yunyu Zhou 2016[28]	30	30	18/12	17/13	60.40±5.20	61.50±5.10	-	-
Yingjie Li 2016[29]	60	60	38/22	36/24	73.3±3.5	72.7±3.6	-	-
Yihao Wang 2016 [30]	48	48	60/36		62.30±5.40		-	-
Yongjie Qiao 2016[31]	40	40	28/12	26/14	52.36±12.67	53.06±13.04	7. 89±2. 86	8. 17±2. 44
Lingzhun Wang 2016[32]	30	30	37/23		65.12±9.760		5.35±1.56	
Qicai Fan 2015[33]	48	42	30/18	28/14	60.80±5.80	62.30±8.60	-	-
Wei Xu 2015[34]	46	46	29/17	31/15	65.89±7.21	66.14±6.58	5.72±3.96	6.41±4.08
Hengdong Liu 2015[35]	33	33	18/15	17/16	70.30±2.30	70.50±2.10	5.50± 1.20	5.4 0± 1.30
Zhimei Huang 2015[36]	90	78	48/42	42/36	74.20±4.10	73.70±4.30	3.30± 1.20	3.50± 1.50
Jing Li 2015[37]	55	55	38/17	36/19	56.58±6.36	57.32±6.33	5.23±0.78	5.41±0.72
Lei Xie 2014[38]	40	40	23/17	25/15	46.00±87.00	42.00±86.00	-	-
Ting Li 2014[39]	85	52	52/33	37/15	68.20±2.40	64.90±3.20	13.70±1.50	11.50±1.80
Hongbin Cai 2013[40]	34	35	17/17	20/15	56.79±5.89	57.02±7.50	12.77±5.30	11.89±4.39
Yongping Hu 2012[41]	35	35	17/18	19/17	55.54±5.42	52.46±5.01	-	-
Tingzhu Wu 2010[42]	43	37	48/32		65.10±10.40		-	-

**Table 2:** The feature Table 2 of meta-analysis.

Study	Type of disease	Dose of Clopidogrel (mg/day)	The species of Danshen	Dose of Danshen (/day)	Time of treatment (month)	Inclusion Criteria	Evaluation criteria for curative effect	Observation index
Qing Shi 2018	UAP	50	Salvianolate	*❷*	2	11)	(3)	①④⑥⑦⑨
Wenjun Gao 2018	CHD	75	Salvianolate	*❸*	0.5	11)	(8)	①⑥⑦④
Chunyan Xue 2017	CHD	75	STS	*❺*	1	3)	(1)	①③⑦
Guangzhi Li 2017	CHD	75	CDDP	*❶*	3	11)	(3) (2)	①②⑦
Jianhua Guo 2016	CHD	75	CDDP	*❶*	0.5	7)	(3)	①②
Jing Feng 2016	UAP	75	DALHI	*❹*	0.5	2)8)	(2)	①②⑥
Qianfan Zhao 2016	CHD	75	DALHI	*❹*	6	6)	(1)(2)	②③⑤⑥
Yunyu Zhou 2016	SAP	75	STS	*⓭*	1	3)	(1)	①③⑤⑦
Yingjie Li 2016	UAP	75	CDDP	*❶*	0.2	5)	(3)	①⑥
Yihao Wang 2016	CHD	75	Salvianolate	*⓬*	0.75	11)	(5)	①⑥
Yongjie Qiao 2016	CHD	75	CDDP	*❶*	6	11)	(5)	①⑦⑧⑨
Lingzhun Wang 2016	CHD	75	Danshen tablet	*⓫*	12	11)	(8)	①⑥⑦
Qicai Fan 2015	CHD	75	CDDP	*❶*	3	2)	(2)	①②
Wei Xu 2015	CHD	150	CDDP	*❿*	1	1)2)	(1)	①⑥
Hengdong Liu 2015	UAP	75	Salvianolate	*⓬*	1	4)	(3)	①⑥
Zhimei Huang 2015	CHD	75	CDDP	*❶*	3	11)	(8)	①⑥
Jing Li 2015	CHD	75	CDDP	*❾*	1	6) 9)	(7)	①③⑥⑨
Lei Xie 2014	UAP	75	GDDP	*❽*	2	4)	(3)	①②⑥
Ting Li 2014	CHD	75	CDDP	*❶*	3	10)	(3)	①⑥
Hongbin Cai 2013	UAP	75	CDDP	*❼*	0.75	1)5)	(4)	①②⑦
Yongping Hu 2012	UAP	75	STS	*❻*	0.5	2)	(3)	②③
Tingzhu Wu 2010	CHD	75	DALHI	*❹*	0.5	2)	(8)	①

*Type of disease:* Unstable angina pectoris: UAP; Stable angina pectoris: SAP.

*The species of Danshen:* Compound Danshen dropping pills: CDDP; Danshen and Ligustrazine Hydrochloride Injection: DALHI; Guanxin Danshen Dropping Pill: GDDP.

*Dose of Danshen*:*❶*270mg/times, 3 times, *❷*200 mg+5% glucose solution, 250ml, i.v., *❸*200 mg+ glucose solution, 200ml, i.v., *❹*10ml + 0.9% sodium chloride 250ml, i.v., *❺*60mg + 5% glucose solution, 250ml, i.v., *❻*40mg, i.v., *❼*270mg/times, 2 times, *❽*400mg/times, 3 times, *❾*7.29g/times, 3 times, *❿*125mg/ times, 3 times, *⓫*3 pieces /times, 3 times, *⓬*200mg, *⓭*60mg.

*Inclusion Criteria*: 1) Chinese medicine clinical research guiding principles. 2) Criteria for naming and diagnosis of ischemic heart disease. 3) A guide to diagnosis and treatment of chronic stable cardiac arrest in China. 4) Clinical diagnostic criteria for unstable angina pectoris. 5) Diagnosis and treatment of unstable angina pectoris. 6) Internal medicine. 7) Guidelines for the diagnosis and treatment of coronary heart disease in the Chinese medical association for cardiovascular diseases. 8) Medicine of traditional Chinese medicine. 9) Standard of TCM syndrome diagnosis. 10) Guidelines for the diagnosis and treatment of coronary heart disease. 11) Other.

*Evaluation criteria for curative effect:* (1) Guiding principles of clinical research on new drugs of traditional Chinese medicine. (2) Evaluation criteria of angina pectoris and electrocardiogram effect in coronary heart disease. (3) The number of episodes of angina pectoris, excellence: reduce over 80%, effective: reduce over 50% and ineffective: reduce under 50%. (4) Practice of internal medicine. (5) Guiding principles of clinical research on cardiovascular system drugs. (6) Evaluation criteria of angina pectoris and electrocardiogram effect in coronary heart disease. (7) Medicine. (8) Other.

*Observation index*: ① Clinical total efficiency. ② Electrocardiogram effect. ③ Frequency and duration of angina pectoris. ④ Vascular function. ⑤ Dosage of nitroglycerin. ⑥ Adverse reaction. ⑦ Blood index. ⑧ Clinical symptom improvement time. ⑨ Heart function.

**Table 3:** Inhibition ( IC_50_) of Danshen's effective components on different CYP450 isozymes in human liver microsomes.

Components	type of Probe	IC_50_ ( *μ*M )										References
		CYP1A2	CYP2B6	CYP2C9	CYP2C9*∗* 1	CYP2C9*∗* 2	CYP2C9*∗* 3	CYP2C19	CYP2D6	CYP3A4	P450 2J2	
Tanshinone IIA	1, 3 - 7, 9	1.3- 10.10	-	> 100	-	-	-	> 100	13.47, > 200	> 100	2.5	[43–47]
Cryptotanshinone	1, 3 - 7, 10	0.75 - 3.06	-	23.86-33	1. 74	2. 64	3. 17	> 100	75	> 100	-	[43, 44, 47, 48]
Miltirone	1, 4 - 6, 8	1.73	-	8.61	-	-	-	26.9	30.20	33.88	-	[43, 49]
Dihydrotanshinone	1, 2, 4 - 8, 10	0.5- 2.25	2. 64	7.48	0. 19	0. 56	1. 52	0.6	11.70-35.4	0.367-3.22	-	[43–45, 48, 50]
Tanshinone I	1, 3 - 7	0.75-11.61	-	> 100	-	-	-	> 100	> 100	> 100	-	[43–45, 47]
Salvianolic acid A	1, 5	5. 37	-	-	-	-	-	-	11. 53	-	-	[45]
Salvianolic acid B	1, 3, 5 - 7	> 100	-	> 200	-	-	-	-	> 200	12. 35 > 200	-	[45, 47]
Rosmarinic acid	1, 7	10. 32	-	-	-	-	-	-	-	5. 43	-	[45]
Danshensu	1, 3, 5 - 7	> 100	-	50	-	-	-	-	> 200	> 200	-	[47]

-, currently not available.

1: Phenacetin; 2: Mephenytoin; 3: Diclofenac; 4: Tolbutamide; 5: Dextromethorphan; 6: Testosterone; 7: Midazolam; 8: Omeprazole; 9: Astemizole; 10: Fluvastatin.

## Abbreviations

CHD: Coronary heart disease
CES1: Carboxylesterase 1
CYP450: Cytochrome P450
RCTs: Randomized controlled trials
OR: Odds ratio
CI: Confidence interval
WMD: Weighted mean difference
ECG: Electrocardiogram.
